# Estimation of Synthetic Rubber Lifespan Based on Ozone Accelerated Aging Tests

**DOI:** 10.3390/polym17060819

**Published:** 2025-03-20

**Authors:** Jeongnam Kim, Youngki Kim, Youhee Cho

**Affiliations:** Department of Reliability, Virtual Engineering Platform Research Division, Korea Institute of Machinery & Materials, Daejeon 34103, Republic of Korea; kjnnoah@kimm.re.kr

**Keywords:** exposure test, rubber degradation, hardness prediction, linear regression analysis

## Abstract

This study investigates the impact of ozone exposure on the hardness of synthetic rubber specimens (a blend of NR (natural rubber) and CR (chloroprene rubber)) through accelerated aging tests. Using a linear regression model, the research predicts the lifespan of rubber under real-world conditions and demonstrates how established experimental methods can yield novel insights when applied to synthetic rubber. The results show that ozone exposure significantly increases hardness within the first 10 days, stabilizing after day 12. Through analysis, this study calculates acceleration factors based on ozone concentration and temperature, estimating the practical lifespan of synthetic rubber under actual conditions to be approximately 25.76 years. These findings provide valuable indicators for evaluating the durability of synthetic rubber materials and predicting the longevity of rubber products in industrial applications. Furthermore, the research emphasizes the potential for improving lifespan prediction accuracy by incorporating non-linear models or machine learning approaches.

## 1. Introduction

Rubber pads are vital in industrial applications due to their flexibility, durability, and resistance to environmental factors. However, ozone exposure, known as ozone aging, degrades their performance. This process can reduce the service life of rubber pads and impact their reliability. As industries aim to improve machinery longevity and efficiency, researching and mitigating ozone effects on rubber pads has become a priority.

Ozone aging occurs when ozone molecules react with the double bonds in rubber polymers. This reaction breaks the bonds, leading to a deterioration in the material’s physical properties. This deterioration not only compromises the mechanical strength but also the elasticity and shock absorption capabilities of the rubber, which are essential for its intended industrial applications [1,2,3,4].

Treib et al. investigated the effects of ozone exposure on natural rubber and compared the effects on unprotected rubber and rubber treated with ozone inhibitors. This highlights the important protective effect of these antioxidants against ozone-induced degradation, and through various experiments, including tensile tests and IR (Infrared) spectroscopy, we have shown how they mitigate surface cracking and molecular changes caused by ozone exposure [1]. R.G. Newton stated that exposure cracking is cracking that occurs in rubber stretched in an ozone-containing environment, that the rate of crack formation and growth varies with strain, and that neoprene and Thiokol-FA rubber have relatively high resistance. Also, the study reported that light acts to delay crack formation and that cracks grow by merging small cracks [2]. Poikelispää M. et al. showed that partial replacement of carbon black (CB) with nanodiamond (ND) improved the tensile strength, wear resistance, and dynamic properties of natural rubber–butadiene rubber composites, which has useful performance-enhancing effects, especially in the tire and mining industries [3]. Hara J. et al. confirmed that adding graphene oxide (GO) modified with p-phenylenediamine (PPD) to nitrile rubber (NBR) significantly improved thermal oxidation stability by strengthening intermolecular interactions and also improved mechanical properties and durability [4]. Kamaruudin et al. highlight the susceptibility of rubber to ozone-induced stress cracking, particularly how ozone exposure reduces the appearance and usability of rubber. The study also highlights the protective role of water against ozone attack, showing how immersion in water can substantially mitigate the formation of cracks on rubber surfaces exposed to ozone exposure [5]. Zheng et al. showed that ozone aging has a significant impact on the surface morphology, molecular structure, and mechanical properties of natural rubber, with crack size increasing over time. In addition, squalene, a compound with a structure similar to natural rubber, simulated the ozone aging process and showed that it could lead to molecular changes and the formation of specific compounds and free radicals during aging [6]. Guo et al. presented a method for predicting the shelf life of rubber products based on step-stress accelerated aging experiments and an intelligent algorithm. Through this study, acceleration factors were estimated by considering the non-Arrhenius phenomenon during the rubber aging process, and the lifespan of rubber pads was predicted using this [7,8]. Moon et al. performed swelling tests on aged rubber compounds, summarized the relationship between strain energy density and crosslink density in a formula and proposed a method to predict the aging behavior of NR (natural rubber)/BR (Butadiene Rubber) blends using crosslink density [9]. Lake found that ozone crack growth in rubber requires a critical level of strain, beyond which the rate of growth is significantly affected by ozone concentration rather than strain, with added complexity caused by factors such as gas flow and test piece size. We investigated whether chemical anti-ozonants can form a protective layer on rubber to prevent cracking, and their effectiveness may vary depending on the rate of development of the layer compared to the rate of ozone attack [10]. Liu et al. enhanced the prediction of rubber component life and maintenance cycles by introducing modified particle swarm optimization (MPSO) algorithms for the efficient and accurate identification of acceleration factors in rubber-accelerated aging tests. Their approach, utilizing time–temperature equivalence for parameter identification, accurately predicts rubber degradation at room temperature, closely aligning with actual measured results [11].

Ik-Sik Kim et al. investigated the ozone oxidation characteristics of NR using image analysis and FT-IR and confirmed that oxidation starts at carbon-carbon double bonds upon ozone exposure, and carbonyl compounds are generated through continuous chain scission. In addition, image analysis observed the occurrence of numerous long, thin cracks arranged at regular intervals. Caroline et al. observed cracks and hardened areas in NR rubber samples under test conditions of 0.75 ppm, 96 h, and 20% strain. The darkened areas were found to be zones damaged by ozone, resulting in surface discoloration. Mingzhe Lv et al. noted that surface changes in NR are caused by ultraviolet light and ozone oxidation, which damage the polymer structure, leading to discoloration or frosting effects [1,12,13].

This study conducts ozone aging experiments on industrial-sized rubber pads and evaluates their performance degradation in order to thoroughly understand the deterioration process of rubber due to ozone exposure. Ozone reacts with the C=C double bonds in rubber, causing the cleavage of macromolecules. This process accelerates the chemical degradation of rubber due to ozone and promotes the formation and propagation of cracks. Exposure to ozone results in the relaxation of internal stress within the rubber, leading to changes in its mechanical properties. This effect is significantly influenced by the ozone concentration, the thickness of the sample, and the degree of strain applied. Notably, higher concentrations of ozone increase the rate of stress relaxation and the growth of cracks. Rubber products exposed to external environments are at risk of rapid damage, starting with crack formation due to ozone. Unstable initial ozonide formed by the reaction of ozone with rubber decompose into ozone decomposition elements containing carbonyl groups, and these rearrange to form new ozonide structures, leading to a fully cross-linked and altered state of the rubber’s double bonds [14,15,16,17].

The purpose of this study is to quantitatively analyze the effect of ozone exposure on the hardness of synthetic rubber specimens through accelerated aging tests and to predict their lifespan under real-world conditions using a linear regression model. It demonstrates how applying established experimental and analytical methods to synthetic rubber (NR and CR (chloroprene rubber) blend) can yield novel insights.

This study focuses on modeling the patterns of hardness changes in synthetic rubber used for industrial purposes and identifying the stabilization point where the hardness reaches a steady state. Such analyses serve as crucial indicators for evaluating the durability of synthetic rubber materials, whose characteristics are less well-known and predicting the lifespan of rubber products. Additionally, by calculating acceleration factors based on experimental conditions, such as ozone concentration and temperature, and applying them to real-world scenarios, the study estimates the practical lifespan of synthetic rubber.

## 2. Materials and Methods

### 2.1. Specimen Production

**Figure 1:**
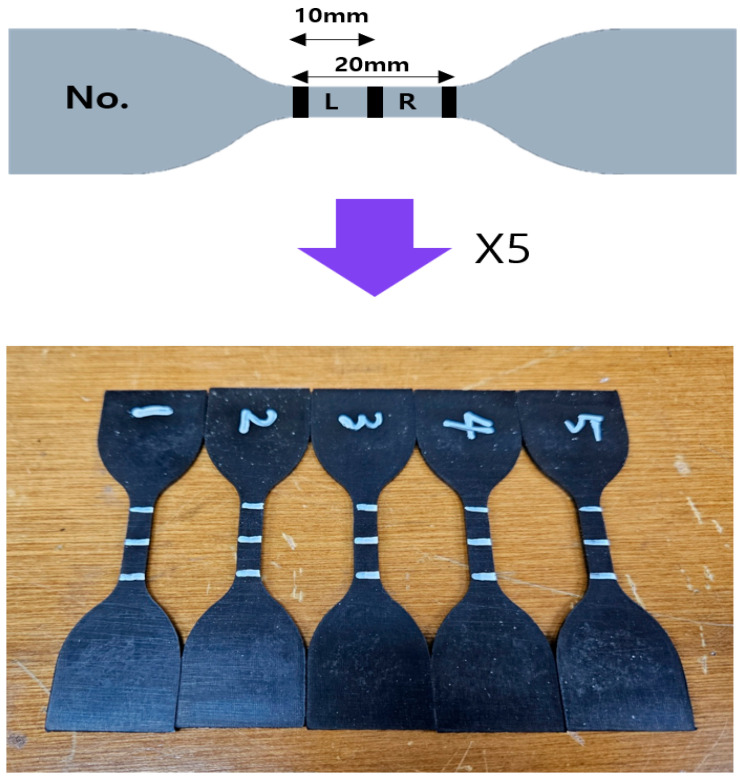
The standard rubber specimens.

The standard rubber specimen shown in Figure 1 is manufactured and consists of a synthetic mixture of NR (natural rubber) and CR (chloroprene rubber). This material was developed for pads installed on harbor walls to secure ships to the shoreline for long periods of time. Environmental factors in the harbor include weather (temperature), oxidation, and ozone. This synthetic rubber offers superior thermal stability compared to natural rubber and exhibits superior resistance to oil and related compounds. It is also suitable for improving resistance to oxidizing agents, oxygen, and ozone, which can shorten the product life [14]. The chemical composition of the manufactured rubber specimen is shown in Table 1.

### 2.2. Ozone Exposure Test and Hardness Data Collection

The ozone test was conducted on five standard rubber specimens (Figure 1), not tensile specimens, per KS M 6518 (2021) [18]. The chosen conditions (0.5 ppm ozone, 50 °C) reflect accelerated aging relevant to harbor pad exposure. The specimens were exposed to ozone at a concentration of 0.5 ppm and a temperature of 50 °C for 9 days. Observations of the specimen surfaces were made every 24 h using a microscope, and hardness was measured using an A Shore hardness tester. Measurements began at least 5 mm away from the specimen’s edge, maintaining a minimum spacing of 12 mm between points. The durometer’s indenter was pressed perpendicularly against the specimen with a constant force and held for 15 s before reading the value. To ensure consistency, at least five measurements were taken per specimen, and the median value was calculated to determine the final hardness. Each specimen was divided into left and right areas from the center, as shown in Figure 1, for comparative observation. The hardness of each area of the same specimen was also measured. The ozone exposure test was conducted in a controlled chamber using an ozone generator capable of maintaining a stable concentration of 0.5 ppm at 50 °C. Ozone levels were continuously monitored using a calibrated UV-based ozone analyzer with an accuracy of ±0.01 ppm. The analyzer was calibrated daily using a reference standard gas mixture (0.5 ppm O_3_ in N_2_) to ensure measurement reliability. Measurements were recorded every 12 h, and any deviations beyond ±5% of the target concentration were corrected by adjusting the ozone generator output. This rigorous monitoring and adjustment protocol ensured stable and reproducible test conditions throughout the 9-day experiment.

### 2.3. Data Analysis

Through this procedure, the hardness data of the rubber specimens’ surfaces over time were collected. Regression analysis was then conducted to identify trends in the data and perform predictions under the experimental conditions. By conducting linear regression analysis on the hardness change data of the rubber specimens, the relationship between ozone exposure time and hardness can be modeled to predict the lifespan of the specimens. This is crucial for understanding the rate of hardness change and evaluating the durability of the specimens. Linear regression models the relationship between the independent variable *x* and the dependent variable *y* as a linear function, which is expressed as follows:

(1) $$y=\beta_{0}+\beta_{1}x+\epsilon$$

where *y* is hardness, *x* is ozone exposure time, $\beta_{0}$ is the initial value of hardness, $\beta_{1}$ is the rate of change in hardness over time, and ϵ is the error term.

The regression coefficients $\beta_{0}$ and $\beta_{1}$ are estimated using the least squares method, which minimizes the sum of the squared distances (residuals) between the actual data points and the regression line. The model’s fit is evaluated using the coefficient of determination, R^2^. R^2^ ranges from 0 to 1, with values closer to 1 indicating a better fit of the model to the data [19]. The regression model can be used to predict the time ($x_{p}$) at which hardness falls below a certain threshold. For example, the equation to predict the time at which hardness falls below 50 is as follows:

(2) $$x_{p}=\frac{y_{th}-\beta_{0}}{\beta_{1}}$$

here, $y_{th}$ is the specified threshold hardness value. The Pearson correlation coefficient γ is used to evaluate the linear relationship between ozone exposure time and hardness. The value of γ ranges from −1 to 1, where 1 indicates a perfect positive linear relationship, −1 indicates a perfect negative linear relationship, and 0 indicates no linear relationship [20].

By using both R^2^ and *γ*, the model’s goodness of fit and the linear relationship between variables can be more accurately evaluated, providing a more robust interpretation of the results.

### 2.4. Life Time Calculation

For the acceleration test conditions, the ozone concentration is 0.5 ppm, and the temperature is 50 °C. For the actual coastal conditions, the typical ozone concentration is 0.02 ppm (average atmospheric ozone level), and the average temperature is 25 °C.

Ozone concentration has a significant effect on the reaction rate, and the acceleration factor due to ozone concentration can be expressed as follows:

(3) $${AF}_{ozone}=\frac{Ozone\;concentration\;under\;accelerated\;conditions\;}{Ozone\;concentration\;under\;actual\;conditions}$$

By substituting these values into the Equation (3), ${AF}_{ozone}=25$. This indicates that the service life under actual conditions will be extended by a factor of 25 when the ozone concentration is reduced from 0.5 ppm to 0.02 ppm.

In this study, the Arrhenius equation was applied to predict the lifespan of rubber materials by deriving acceleration factors based on temperature and ozone concentration, as this method is suitable for estimating long-term degradation trends within a relatively short experimental period. Although mechanical loading and cyclic deformation can significantly influence rubber degradation in real-world conditions, these factors were not included in this study because the experimental design and analysis were focused on quantitatively assessing the effects of ozone aging. The acceleration factor due to temperature can be calculated using the Arrhenius equation:

(4) $${AF}_{temperature}=e^{\frac{E_{a}}{k}(\frac{1}{T_{actual}}-\frac{1}{T_{accelerated}})}$$

where $E_{a}=0.73\;eV$ is the activation energy for ozone degradation of rubber [21]. $k=8.6171\times \frac{10^{-5}\;eV}{K}$ is the Boltzmann constant. $T_{actual}=298\;K$ is the actual temperature. $T_{accelerated}=323\;K$ is the accelerated test temperature. By substituting these values into the Equation (4), ${AF}_{temperature}\approx 9.0278$. The total acceleration factor is determined by multiplying the individual factors for ozone concentration and temperature:

(5) $${AF}_{total}={AF}_{ozone}\times {AF}_{temperature}=225.695$$

Thus, the total acceleration factor that the predicted life time under accelerated test conditions is approximately 53.25 times longer than under actual conditions. After verifying the expected lifetime under accelerated test conditions in actual experiments, the actual life can be calculated using the total acceleration factor:

(6) $$L_{actual}={AF}_{total}\times L_{accelerated}$$

## 3. Results

### 3.1. Standard Specimen Results for 9 Days

**Figure 2:**
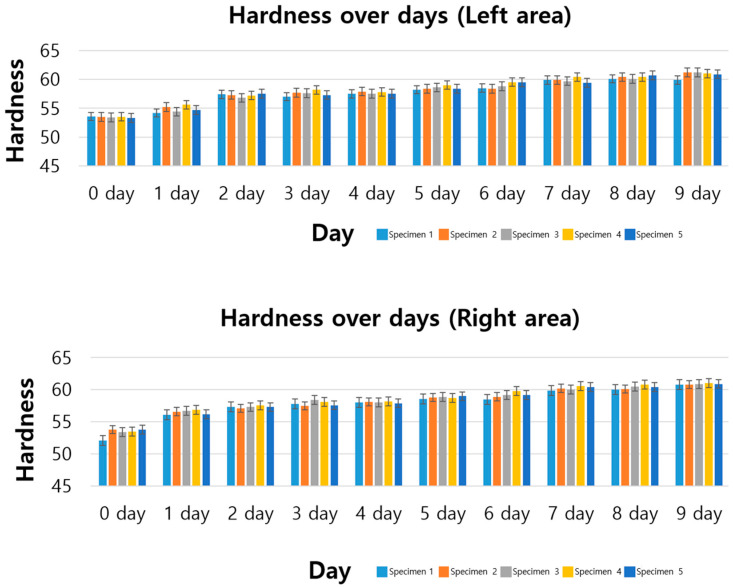
Hardness data (Shore A) graphs and tables for standard specimens (9 days).

In this study, changes in hardness due to ozone exposure were measured for synthetic rubber specimens. The specimen was divided into two sections, left and right, based on the center, and the changes in hardness were compared. Figure 2 shows the change in hardness from day 0 to day 9.

The hardness change in the left area is shown in the figure below. Hardness increased slightly from day 0 to day 1, the initial stage of ozone exposure, but a sharp increase in hardness was observed after day 2. In particular, on the 9th day, the average hardness increased to 60.8. This is a 13.8% increase compared to the initial average.

The hardness changes in the right area showed a similar trend to the left area. Hardness increased slightly from the initial 0 to 1 day, and a rapid increase in hardness was confirmed from the 2nd day. The average hardness on the 9th day was 60.9, an increase of 14.2% compared to the initial hardness.

**Figure 3:**
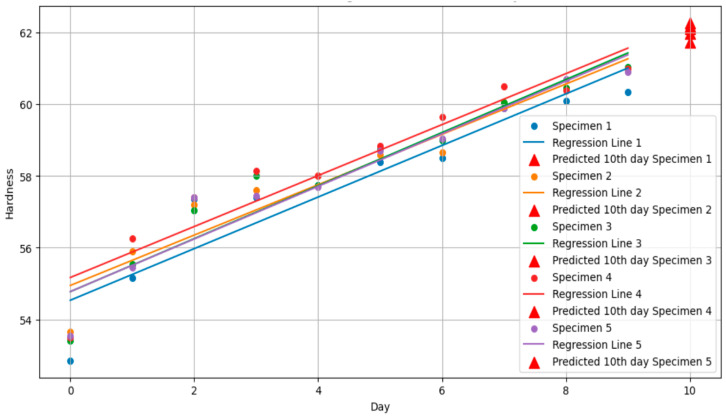
Hardness (Shore A) over time with regression lines and 10th day prediction.

Linear regression analysis was performed to analyze the change in hardness of rubber specimens due to ozone exposure. The results are shown in Figure 3 and Table 2.

Linear regression analysis of the hardness change data yielded the hardness change rate and initial hardness value for each specimen, as summarized in Table 2. The analysis revealed a distinct pattern: hardness increased rapidly within the first few days (e.g., a sharp rise from day 1 to day 2), followed by a slower increase until day 9. The regression results (Table 2) show slope and intercept values for each specimen, with coefficient of determination (R^2^) values ranging from 0.87 to 0.92, indicating a strong model fit.

The following conclusions can be drawn from the graph and regression analysis results:

1. Increase in hardness: In all specimens, hardness tended to increase as ozone exposure time increased;
2. Model suitability: The coefficient of determination (R^2^) value is between 0.87 and 0.92, confirming that the linear regression model explains the hardness change data well;
3. Pearson correlation coefficient evaluation: Pearson correlation coefficient was calculated to evaluate the linear relationship between ozone exposure time and hardness, and a positive correlation was found in all specimens. The values shown in the table are all between 0.94 and 0.96, showing a very high positive correlation. This indicates that there is a strong positive linear relationship between ozone exposure time and hardness change, meaning that as the exposure time increases, the hardness also steadily increases.

### 3.2. Standard Specimen Results for 10th Day

**Figure 4:**
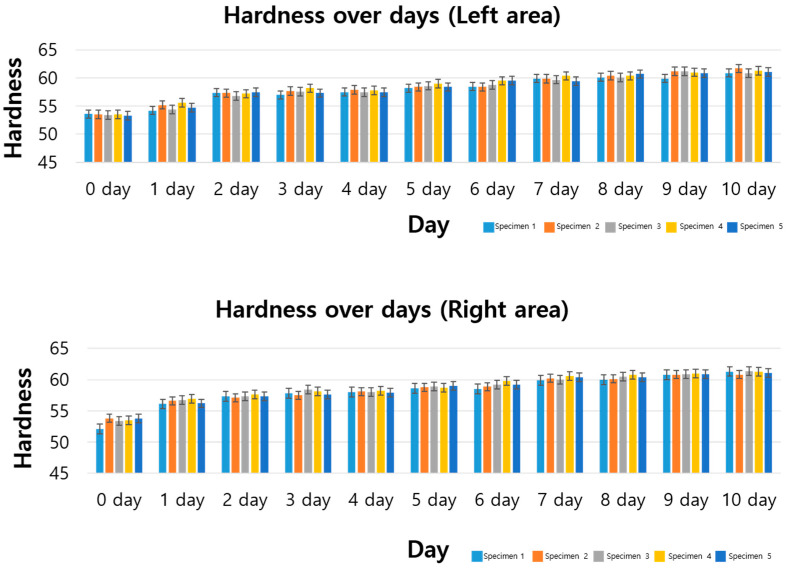
Hardness data (Shore A) graphs and tables for standard specimens (10 days).

Additionally, the experiment was conducted until the 10th day, and the data are shown in Figure 4. In the left area, the average hardness value on the 10th day was 61.2, 12.6% higher than the initial value, and increased by 0.5% compared to the 9th day. Similar trends were observed in the right area, with an average hardness of 61.2, a 14.8% increase from the starting day. The minor differences between predicted and actual hardness values (Table 3) may be attributed to experimental variability, such as slight fluctuations in ozone concentration or temperature, and the assumption of linearity in the regression model, which may not fully capture subtle non-linear degradation effects at later stages. These discrepancies, however, remain within a 2% error margin, indicating robust model performance. In the right area, the average hardness value on the 10th day was 61.2, which was about 14.8% higher than the hardness value on the starting day.

The comparison between predicted and actual values is summarized in Table 2. The error value was less than 2%, and it was confirmed that the predictions were made at a relatively similar level. Therefore, considering the experimental conditions and data variability, it can be judged to be within a reasonable error range.

### 3.3. Standard Specimen Results for 16 Days

**Figure 5:**
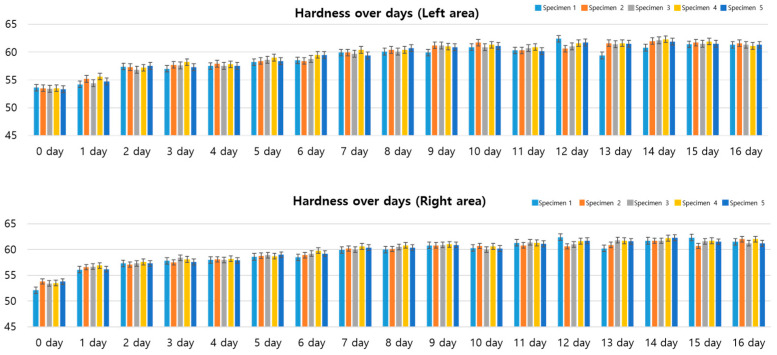
Hardness data (Shore A) graphs and tables for standard specimens (16 days).

As shown in Figure 5, the experiment was conducted until the 16th day, and after the 12th day, the hardness remained constant with almost no change. This stabilization likely occurs due to the saturation of ozone-reactive sites, particularly the carbon-carbon double bonds (C=C) in the NR/CR blend, which are primary targets for ozone attack [14]. Once these reactive sites are fully oxidized, further ozone exposure does not significantly alter the surface hardness as the degradation process reaches a plateau. This observation aligns with findings by Razumovskii et al. [14], who noted that ozone-induced stress relaxation in rubber slows as reactive sites are depleted. This stabilization suggests that the rubber achieves a steady-state resistance to further ozone degradation, which has implications for its long-term durability in industrial applications. This suggests that the rubber specimen reached a stable state in which the hardness change due to ozone exposure was similar. This hardness change was similar in both the left and right areas, and no significant difference was observed between the two areas. Therefore, the hardness changes due to ozone exposure showed a pattern of rapidly increasing at the beginning of the experiment and stabilizing after the 12th day, indicating that the rubber material reached a stable state after experiencing a certain hardness change in an ozone environment.

## 4. Discussion

This study quantitatively analyzed the change in the hardness of rubber due to ozone exposure using accelerated aging tests. The results, including an expected life of 25.76 years under coastal conditions (0.02 ppm ozone, 25 °C), are directly relevant to the marine industry where rubber pads secure ships. The model can guide the design of durable components to reduce costs and increase reliability. Although mechanical loading was excluded due to experimental complexity, future applications could extend the use of seals not only in the marine industry but also in automotive, construction, or aerospace, where they face ozone exposure. Therefore, this study primarily analyzes the chemical degradation mechanisms caused by temperature and ozone exposure, leaving the incorporation of mechanical loading and deformation effects for future research to develop a more comprehensive lifespan prediction model. The results of modeling the hardness changes in each specimen using linear regression analysis demonstrated that the laboratory data could be effectively used to predict hardness changes in real-world environments. In particular, the data collected after 12 days, when the hardness stabilized, provided important insights suggesting that rubber materials can maintain some resistance to ozone, thereby extending their lifespan.

However, although the error rate between the experimental results and the predictive model was found to be within 2%, this may have been caused by variable differences between the laboratory conditions and real-world environments. For example, while the ozone concentration and temperature were consistently maintained in the laboratory, these factors are likely to fluctuate in real-world conditions. Considering these differences, it may be necessary to incorporate calibration steps that reflect various environmental variables for practical applications of the model.

In addition, while this study used linear regression analysis to predict hardness changes, more precise prediction models could be built by incorporating non-linear models or machine learning-based methods. Such follow-up research is expected to further improve the accuracy of rubber material lifespan predictions.

**Figure 6:**
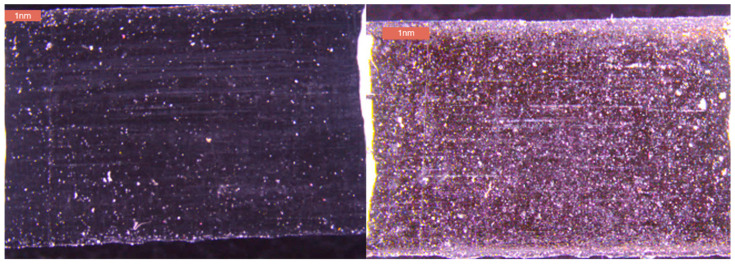
Micrographs of standard rubber specimens. (**Left**) Surface before experiment. (**Right**) Surface after 12 days.

Moreover, although this study primarily analyzed rubber degradation based on hardness changes, as shown in Figure 6, it was observed through microscope images that surface discoloration due to ozone exposure could also serve as an important indicator of degradation. Figure 6 compares the specimen before testing (left) and after 12 days (right), showing clear surface discoloration and minor cracking due to ozone exposure. These observations, supplementary to our hardness focus, suggest oxidation, with cracks appearing as thin, evenly spaced lines, reinforcing the degradation mechanism. This indicates that the surface material reacted with ozone, leading to oxidation, and suggests that chemical degradation due to ozone affects not only the hardness but also the color and structure of the specimen.

Notably, this discoloration could also be linked to overall structural changes in the specimen. While the surface of the pre-experiment specimen was relatively uniform and smooth, the specimen after 12 days exhibited signs of minor cracking and wear. This suggests that exposure to ozone caused the surface organic compounds to deteriorate, resulting in increased hardness and a weakened surface.

## 5. Conclusions

This study analyzed the hardness change of NR/Cr synthetic rubber specimens exposed to ozone and presented a methodology to predict service life in real environments using a linear regression model. According to the experimental results, the hardness of the rubber specimens stabilized after 12 days, and the predicted service life was approximately 25.76 years under real conditions. The model derived through linear regression analysis showed relatively consistent results with the laboratory data and can be an important indicator for evaluating the durability of commercial rubber material pads. However, this prediction model is based solely on experimental data and acceleration factor evaluations. It does not represent an absolute service life value that accounts for the complex interplay of factors such as fatigue and load in real industrial environments. Rather, it provides an estimate of service life, as it lacks the long-term field verification typical of accelerated aging studies. These factors could accelerate crack propagation and material breakdown, potentially reducing the predicted lifespan significantly below 25.76 years in dynamic environments like industrial machinery or harbor settings. The results of this study are important for understanding the degradation of NR/CR synthetic rubber in ozone environments and providing a basic methodology for predicting service life based on experimental data, contributing to an improved understanding of how industrial materials such as synthetic rubber pads change in ozone environments. In addition, the validity of the service life prediction model based on experimental data was proven, suggesting the possibility of real industrial application through service life prediction. However, future studies will need to analyze nonlinear changes in hardness and build predictive models that reflect various environmental factors.

## Figures and Tables

**Table 1:** Chemical composition of the rubber pad.

NR	CR	Carbon Black	CaCO_3_	Others (Ca, Mg, Zn, etc.)
48%	48%	10%	25%	17%

**Table 2:** Results of regression analysis.

Specimen No.	Slope	Intercept	Coefficient of Determination (R^2^)	Pearson Correlation Coefficient (γ)
1	0.72	54.53	0.87	0.94
2	0.70	54.94	0.92	0.96
3	0.74	54.77	0.92	0.96
4	0.71	55.17	0.89	0.94
5	0.73	54.77	0.93	0.96

**Table 3:** Comparison table of predicted and actual hardness (Shore A) values.

	Specimen 1	Specimen 2	Specimen 3	Specimen 4	Specimen 5
Predicted value	61.74	61.98	62.18	62.28	62.12
Actual value	61.30	61.80	61.40	61.30	61.20
Error (%)	0.72	0.002	1.27	1.60	1.50

## Data Availability

The original contributions presented in the study are included in the article; further inquiries can be directed to the corresponding authors.
